# MicroRNA-15b-5p inhibits tumor necrosis factor alpha-induced proliferation, migration, and extracellular matrix production of airway smooth muscle cells via targeting yes-associated protein 1

**DOI:** 10.1080/21655979.2022.2036890

**Published:** 2022-02-16

**Authors:** Shaolin Zeng, Juan Cui, Yunting Zhang, Zhishui Zheng, Jun Meng, Junying Du

**Affiliations:** a Department of Respiratory and Critical Care Medicine, Renmin Hospital of Wuhan University, Wuhan, Hubei Province, China; b Department of Critical Care and Intensive Care Medicine, Xiangzhou District People's Hospital, Xiangyang, Hubei Province, 441100, China; c Department of Pediatrics, Xi’an No. 3 Hospital, Xi’an, Shaanxi Province, China

**Keywords:** Asthma, miR-15b-5p, YAP1, ASM, proliferation, migration, ECM

## Abstract

The excessive proliferation and the deposition of extracellular matrix (ECM) of airway smooth muscle (ASM) cells facilitates airway remodeling in asthma. This study explores how microRNA-15b-5p (miR-15b-5p) functions in modulating the proliferation, migration, inflammatory response, and ECM deposition of ASM cells. MiR-15b-5p and yes-associated protein 1 (YAP1) mRNA expression levels in tumor necrosis factor alpha (TNF-α)-induced ASM cells were, respectively, examined by real-time quantitative polymerase-chain reaction. Besides, the proliferative ability and migrative potential of ASM cells were examined by cell counting kit-8 assay, 5-bromo-2 ‘-deoxyuridine assay, and transwell assays, respectively. Interleukin-6 and interleukin-8 levels in ASM cells were detected by enzyme-linked immunosorbent assay. YAP1, collagen I, and collagen III expressions in ASM cells were detected by Western blot. With dual-luciferase reporter gene assay, the relations between miR-15b-5p and YAP1 3ʹUTR in ASM cells was examined. MiR-15b-5p expression level was reduced in ASM cells treated with TNF-α. MiR-15b-5p repressed TNF-α-initiated growth and migration of ASM cells and also suppressed IL-6 and IL-8 secretion, and inhibited collagen I and collagen III expressions in ASM cells. Furthermore, it was validated that YAP1 was a downstream target of miR-15b-5p in ASM cells. Notably, YAP1 overexpression attenuated the inhibitory effects of miR-15b-5p up-regulation on the proliferation, migration, and inflammatory response, as well as ECM deposition of TNF-α-induced ASM cells. In conclusion, miR-15b-5p/YAP1 axis modulates the growth, migration, inflammatory response, and ECM deposition of ASM cells, thus participating in the pathogenesis of asthma.

## Graphical Abstract

**Figure:**
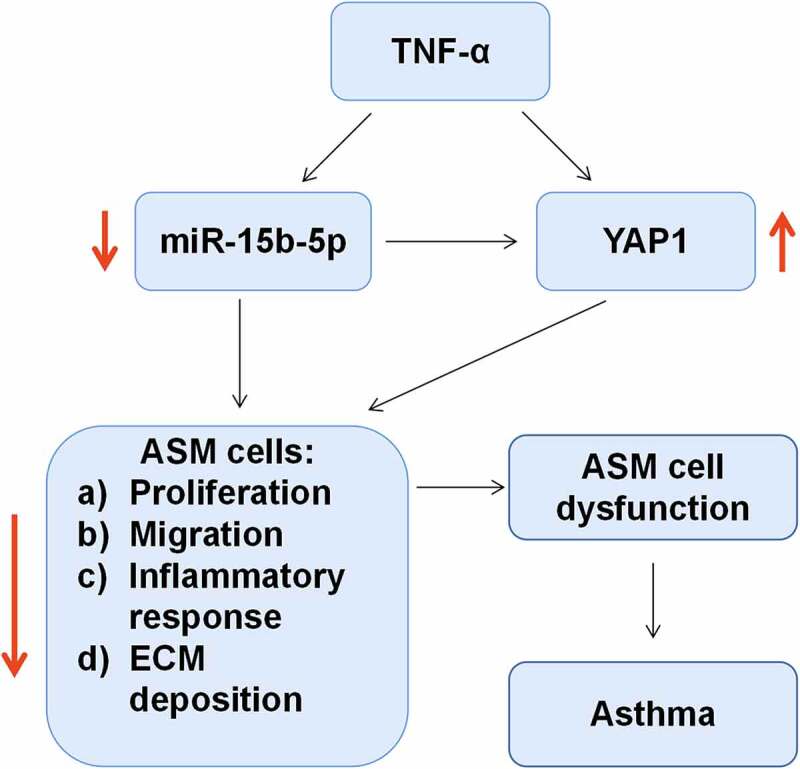

## Introduction

Asthma is a common respiratory disease characterized by chronic airway inflammation, airway hyperresponsiveness (AHR), and airway remodeling, affecting 400 million people worldwide [1,2]. Multiple inflammatory cytokines are pertinent to airway inflammation and AHR in asthma [3]. Among them, tumor necrosis factor α (TNF-α) significantly highly expressed in the lung tissue of asthmatic mice and bronchoalveolar lavage fluid of asthmatic children, has been reported to induce the proliferation, migration, and extracellular matrix (ECM) deposition of airway smooth muscle (ASM) cells [4,5]. ASM cells are the main structural components of the airways, and their excessive proliferation causes airway remodeling and can exacerbate the airway inflammatory response by secreting pro-inflammatory cytokines/chemokines, such as interleukin-6 (IL-6) and interleukin-8 (IL-8) [6]. However, the molecular mechanisms of ASM cell hyperproliferation are not fully clarified.

MicroRNAs (miRNAs/miRs) feature prominently in regulating biological processes such as cell growth, migration, autophagy, and apoptosis [7,8]. Multiple miRNAs, such as miR-221, miR-133a, miR-375, miR-142-3p, miR-146a-5p, miR-26a, miR-31 are important regulators in asthma [4,9–13]. For example, miR-26a, miR-146a, and miR-31 are highly expressed in the lung tissue of asthmatic mice and bronchoalveolar lavage fluid of asthmatic children, and this disorder is involved in the pathogenesis of asthma via regulating inflammatory cytokines contents [4]. MiR-192-5p is lowly expressed in asthmatic mouse lung tissue and alleviates airway remodeling in asthma via targeting matrix metalloproteinases 16 and autophagy-related 7 [14]. Interestingly, it is reported that miR-15b-5p expression was declined in the plasma of asthmatics [15]. However, how miR-15b-5p functions in ASM cells are worth exploring.

Yes-associated protein 1 (YAP1) is a pivotal protein in Hippo pathway and is involved in intracellular signal transduction and transcriptional co-activation of downstream genes [16]. YAP1 is vital in regulating biological processes, including cell proliferation, apoptosis, and differentiation [17]. Importantly, YAP1 is also an important modulator of asthma pathogenesis [11,18]. Fibulin-5 can promote ASM cell growth and migration via modulating Hippo-YAP/TAZ signaling pathway [18]. Additionally, YAP1 enhances the pro-angiogenesis of ASM cells by activating STAT3 and VEGF signaling pathways [11]. However, the interaction of YAP1 with miR-15b-5p in ASM cells has not been elucidated.

In this work, we hypothesized that miR-15b-5p played an important role in TNF-α-induced dysfunction of ASM cells. The main objective of this study was to investigate how miR-15b-5p regulates the proliferation, migration, inflammatory response, and ECM deposition of ASM cells. In our study, we found that miR-15b-5p was inhibited in ASM cells treated by TNF-α, and miR-15b-5p could repress the dysfunction of ASM cells by down-modulating YAP1 expression.

## Methods and materials

### Cell culture

Human primary ASM cells (ATCC PCS-130-011™, Manassas, VA, USA) were subsequently cultured in Dulbecco’s modified Eagles medium/Ham’s F12 (Hyclone, Logan, UT, USA) with 10% fetal bovine serum (FBS; Sigma, St. Louis, MO, USA), 100 unit/mL penicillin, and 100 μg/mL streptomycin (Cellgro, Manassas, VA, USA). Human ASM cells were routinely cultured at 37°C in 5% CO_2_. To establish the cell model of asthma, ASM cells were starved in serum-free medium for 24 h and then subjected to treatment with TNF-α (10 ng/ml; Sigma, St. Louis, MO, USA) for 24 h [19]. ASM cells without TNF-α treatment were set as the blank group.

### RNA extraction and real-time quantitative polymerase chain reaction (RT-qPCR)

Total RNA was extracted using the RNAiso kit (Takara, Dalian, China). RNA was treated with DNase (Solarbio, Beijing, China) before cDNA synthesis. RNA was reversely transcribed into cDNA by the miScript reverse transcription kit (Qiagen, Hilden, Germany) and M-MLV reverse transcriptase kit (Promega, Madison, WI, USA). PCR amplification was accomplished on the ABI7500 Real-Time PCR System (Applied Biosystems; Thermo Fisher Scientific, Inc., Foster City, CA, USA) with the SYBR Green PCR kit (TaKaRa, Dalian, China), with U6 and β-actin as internal controls. The relative expressions of miR-15b-5p and YAP1 mRNA were calculated with 2^−ΔΔCt^ method [20]. The primer sequences are displayed in Table 1.

**Table 1.** Primer Sequences for RT-qPCR

Name	Primer sequences
miR-15b-5p	Forward:5’-TCGGGTAGCACACATAATGG-3’
	Reverse:5’-GTGCAGGGTCCGAGGT-3’
U6	Forward:5’-CTCGCTTCGGAGCACA-3’
	Reverse:5’-AACGCTTCACGAATTTGCGT-3’
YAP1	Forward:5’-ACCCACAGCTCAGCATCTTC-3’
	Reverse:5’-GCTGTGACGTTCATCTGGGA-3’
β-actin	Forward:5’-CCCACACTGTGCCCATCTAC-3′
	Forward:5’-GGAACCGCTCATTGCCAATG-3′

### Cell transfection

GenePharma (Shanghai, China) was the provider of miR-15b-5p mimics, mimics negative control (mimics-NC), pcDNA3.1-YAP1 overexpressing plasmid (YAP1), and empty vector (vector). ASM cells (5 × 10^5^ cells per dish) were transiently transfected with miRNA (final concentration of 50 nM) or plasmid (0.5 μg/mL) by Lipofectamine^TM^ 3000 (Invitrogen, Carlsbad, CA, USA). 24 h later, the ASM cells were harvested for further analysis.

### Cell proliferation assay

Cell counting kit 8 (CCK-8, Dojindo, Kumamoto, Japan) was adopted to assess the proliferation of ASM cells. ASM cells were seeded into 96-well plates at 2 × 10^4^ cells/well and cultured at 37°C overnight. 24 h later, the cells were followingly treated with 10 ng/ml of TNF-α for 24 h. Next, 10 μL of CCK-8 reagent was loaded into each well at different time (24, 48, 72, and 96 h) and then incubated for 2 h at 37°C. Subsequently, the values of optical density (OD)_450 nm_ were assessed by a Multiscan MS spectrophotometer (Labsystems, Stockholm, Sweden).

### 5-bromo-2 ′ -deoxyuridine (BrdU) assay

The viability of ASM cells was also detected with a BrdU kit (Invitrogen, Carlsbad, CA, USA). Briefly, the transfected ASM cells were inoculated into 96-well plates at 2 × 10^3^ cells/well and treated with 10 ng/ml of TNF-α for 24 h. Next, the cells were then rinsed with phosphate buffered solution (PBS; Beyotime, Shanghai, China) and 20 μL of BrdU solution was dripped into each well. The cells were followingly incubated for 6 h at 37°C and fixed with 4% paraformaldehyde for 10 min at ambient temperature. After rinsing with PBS, the above cells were incubated with anti-BrdU antibody (Abcam, Cambridge, Massachusetts, USA) for 8 h at 4°C. After that, the nuclei were then stained with 10 μmol/L Hoechst 33342 (Beyotime, Shanghai, China) for 2 h in darkness. Then, cells were then observed under a fluorescence microscope. Cell proliferation rate = numbers of BrdU-stained positive cells/total number of cells × 100%.

### Transwell assay

Transwell migration assays were accomplished to test the migration ability of ASM cells with Transwell inserts (8 µm pore size, Corning Incorporated, NY, USA). Briefly, 24 h after cell transfection, the cells were treated with 10 ng/ml of TNF-α for 24 h. Then, the cells were immersed twice in PBS and subsequently resuspended in serum-free medium (1 × 10^5^ cells/mL). Moreover, 200 μl of cell suspension was loaded into the upper compartment of each Transwell insert, and 600 μL of medium with 20% FBS was dripped into the lower compartment, and the cells were cultured at 37°C for 24 h. After that, the cells retained in the upper were removed with a cotton swab and the migrated cells on the lower surface of the filter were fixed with 4% formaldehyde (Sigma-Aldrich, Shanghai, China) for 30 min and subsequently stained with 0.5% crystal violet (Sigma-Aldrich, Shanghai, China) for 20 min. Ultimately, the migrated cells were counted in five randomly selected fields (magnification × 250) under a light microscope (Olympus, Tokyo, Japan).

### Enzyme ‐ linked immunosorbent assay (ELISA)

Transfected ASM cells (4 × 10^5^/per well) were inoculated in 12-well plates and treated with 10 ng/ml of TNF-α for 24 h. These cells, together with the medium, were harvested and followingly centrifuged at 1000 × g for 10 min at 4°C. The supernatant was then obtained. Subsequently, IL-6 and IL-8 contents in the samples were measured with the corresponding ELISA kits (TaKaRa, Dalian, China).

### Western blot assay

Total proteins were extracted using the RIPA lysis buffer (Beyotime, Shanghai, China) supplemented with protease inhibitor (PMSF), with the protein concentration quantified by the Bicinchoninic Acid (BCA) Protein Assay Kit (Beyotime, Shanghai, China). Equal amounts of protein samples (20 μg per group) were then separated by sodium dodecyl sulfate-polyacrylamide gel electrophoresis and subsequently transferred onto polyvinylidene fluoride (PVDF) membranes (Millipore, Billerica, MA, USA). Then, the membranes were blocked with 5% skimmed milk for 1 h at 37°C and incubated with primary antibodies anti-YAP1 (1:1000, ab52771, Abcam), anti-collagen I (1:1000, ab270993, Abcam), anti-collagen III (1:1000, ab184993, Abcam) and anti-β-actin (1:1000, ab8226, Abcam) overnight at 4°C. The membranes were then washed with Tris Buffered Saline Tween (TBST), and incubated with horseradish peroxidase (HRP)-conjugated goat anti-rabbit (1:5000, ab205718, Abcam) or goat anti-mouse (1:5000, ab205719, Abcam) secondary antibodies for 1 h at 37°C. Ultimately, the protein bands on the membrane were detected with the Pierce ™ ECL Western blotting substrate (Thermo Fisher Scientific, Waltham, MA, USA) and quantified by Image Lab software (Bio-Rad, Hercules, CA, USA).

### Bioinformatic analysis

The binding sites between miR-15b-5p and YAP1 3′-UTR were predicted by the StarBase database (http://starbase.sysu.edu.cn/) [21].

### Dual-luciferase reporter gene assay

The luciferase reporter vectors carrying the fragments of YAP1 3’-UTR containing wild type (WT) or mutated (MUT) binding sites of miR-15b-5p were available from RiboBio (Guangzhou, China). ASM cells (5 × 10^5^ cells/well) were subsequently inoculated in 24-well plates and cultured for 24 h. Next, the reporter vectors were co-transfected into ASM cells with miR-15b-5p mimics (or mimics-NC) by Lipofectamine^TM^ 3000 (Invitrogen, Carlsbad, CA, USA). 48 h later, the luciferase activity was assessed by a dual-luciferase reporter kit (Promega, Madison, WI, USA), with the renilla luciferase reporter vector as an internal control.

### Statistical analysis

All data analysis was performed by SPSS 18.0 software. All experiments were independently repeated 3 times. Experimental data were displayed in the form of mean ± standard deviation. The differences between two groups were analyzed by student’s *t*-test; one-way ANOVA with post-hoc Tukey test, was used to compare the differences among multiple groups. Statistically, *P* < 0.05 is meaningful.

## Results

This work focused on exploring the expression characteristics of miR-15b-5p and investigating its biological function in regulating ASM cell proliferation, migration, inflammatory response, and ECM deposition, with TNF-α-stimulated ASM cells as a model. In addition, the interaction of miR-15b-5p with YAP1 in ASM cells was also investigated. This study showed that miR-15b-5p could inhibit the proliferation, migration, inflammation, and ECM deposition of TNF-α-induced ASM cells by targeting YAP1.

### TNF-α suppresses miR-15b-5p expression in ASM cells

**Figure 1.**
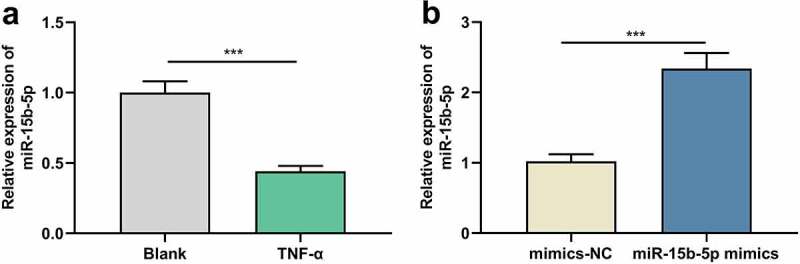
The effects of TNF-α on miR-15b-5p expression in ASM cells.

We first used TNF-α to treat human ASM cells to establish an *in-vitro* asthma model and then detected miR-15b-5p expression in ASM cells by RT-qPCR, revealing that miR-15b-5p expression was inhibited in TNF-α-stimulated ASM cells compared with the blank group (Figure 1(a)). Thus, it might be involved in the TNF-α-induced dysfunction of ASM cells. To delve into the biological role of miR-15b-5p in ASM cells, we transfected ASM cells with miR-15b-5p mimics, along with mimics-NC as a negative control (Figure 1(b)).

ASM cells were treated with TNF-α at a concentration of 10 ng/ml for 24 h, and ASM cells without any treatment were used as a control group (Blank). (a) miR-15b-5p expression in ASM cells was examined by RT-qPCR. (b) The expression of miR-15b-5p in ASM cells transfected with miR-15b-5p mimics or mimics-NC was examined by RT-qPCR. Data are displayed as mean ± standard deviation (n = 3 independent experiments). P < 0.001 versus blank group or mimics-NC group.

### Upregulating miR-15b-5p restrains TNF-α-mediated ASM cell growth and migration

**Figure 2.**
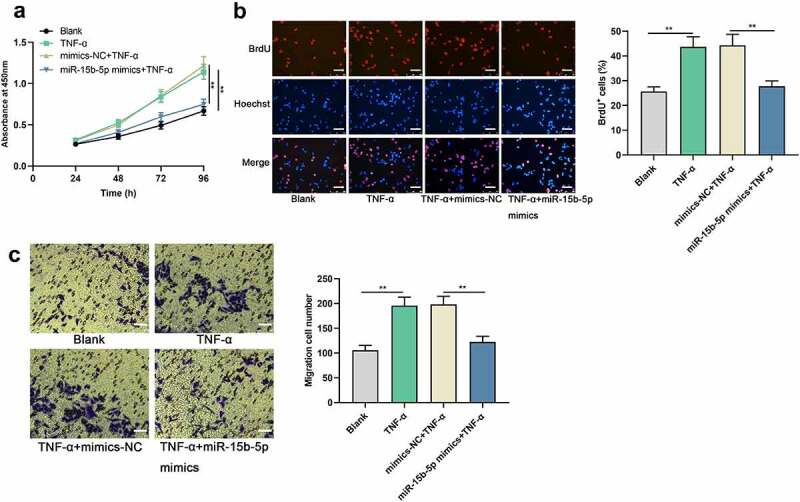
The effects of miR-15b-5p on TNF-α-induced proliferation and migration of ASM cells.

Next, the impact of miR-15b-5p on TNF-α-induced proliferation and migration of ASM cells was investigated. CCK-8, BrdU and Transwell assays suggested that TNF-α stimulation significantly accelerated the proliferation and migration of ASM cell, while miR-15b-5p up-regulation weakened these effects (Figure 2(a-c)). Collectively, miR-15b-5p could probably suppress the dysfunction of ASM cells in asthma.

ASM cells were transfected with miR-15b-5p mimics or mimics-NC, and then TNF-α was used to treat the cells at a concentration of 10 ng/ml for 24 h. (a) CCK-8 assay and (b) BrdU assay were used to examine the effect of overexpression of miR-15b-5p on ASM cell proliferation. Scale bar, 250 μm. (c) Transwell assay was used to examine the effect of overexpression of miR-15b-5p on ASM migration. Scale bar, 250 μm. Data are displayed as mean ± standard deviation (n = 3 independent experiments). *P* < 0.01 versus blank group or mimics-NC + TNF-α group.

### miR-15b-5p up-modulation inhibits TNF-α-induced inflammatory response and ECM deposition

**Figure 3.**
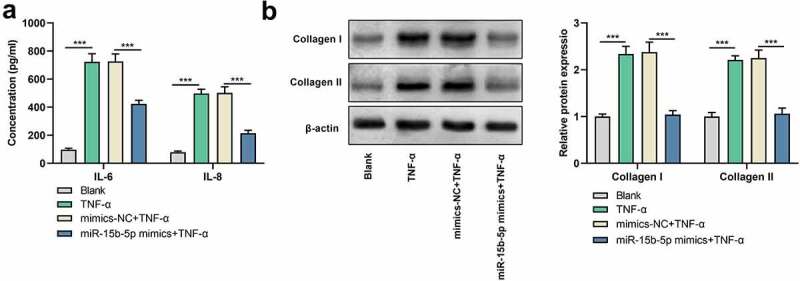
The effects of miR-15b-5p on TNF-α-induced inflammatory response and ECM deposition in ASM cells.

To figure out the impact of miR-15b-5p on the inflammatory response induced by TNF-α, we performed ELISA and observed that IL-6 and IL-8 levels were markedly elevated in ASM cells treated with TNF-α, while those were declined followed by transfecting with miR-15b-5p mimics (Figure 3(a)). Additionally, Western blot indicated that the expression levels of collagen I and collagen III proteins were markedly up-regulated in ASM cells in the TNF-α treatment group as against the blank group, while transfecting with miR-15b-5p mimics significantly decreased those levels as against the TNF-α + mimics-NC group (Figure 3(b)). Collectively, up-regulation of miR-15b-5p impeded TNF-α-initiated inflammatory response and ECM deposition in ASM cells.

ASM cells were transiently transfected with miR-15b-5p mimic or mimics-NC, and treated with 10 ng/ml of TNF-α for 24 h. (a) The levels of IL-6 and IL-8 in ASM cells were measured by ELISA. (b)The expression of collagen I and collagen III proteins in ASM cells were detected by Western blot assay. Data are displayed as mean ± standard deviation (n = 3 independent experiments). ***P < 0.001 versus blank group or mimics-NC + TNF-α group.

### YAP1 is a direct target of miR-15b-5p in ASM cells

**Figure 4.**
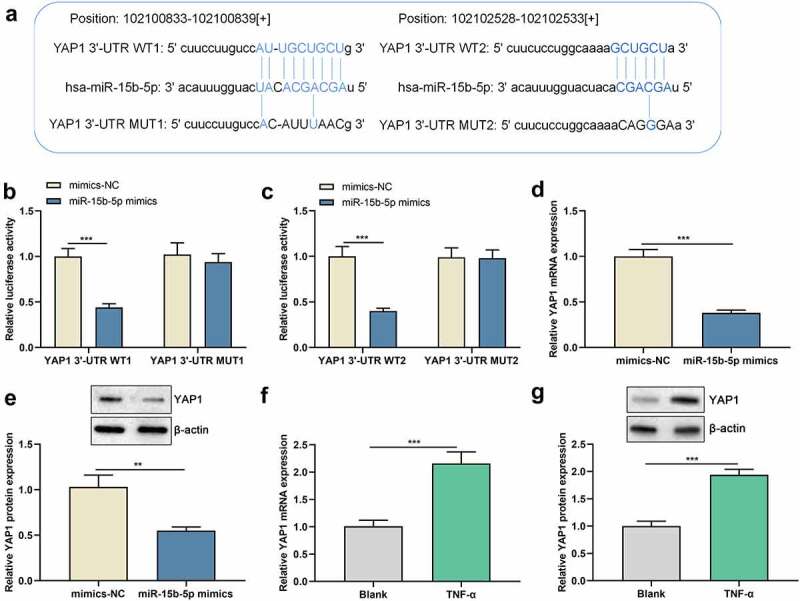
miR-15b-5p directly targets the YAP1 mRNA 3 ′ -UTR.

As to the hidden mechanism of miR-15b-5p in regulating the phenotypes of ASM cells, we conducted bioinformatics analysis and found that the 3’-UTR of YAP1 harbors two binding sites for miR-15b-5p (Figure 4(a)). To clarify whether miR-15b-5p targets YAP1, dual-luciferase reporter gene assay was performed, and it highlighted that miR-15b-5p up-regulation remarkably repressed the luciferase activity of YAP1 3ʹUTR WT1 and WT2, but the luciferase activity of YAP1 3ʹUTR MUT1 and MUT2 were not significantly affected (Figure 4(b and c)). These data suggested that both of the two potential-binding sites were functional. Furthermore, we demonstrated that up-regulation of miR-15b-5p markedly suppressed YAP1 mRNA and protein expression levels (Figure 4(d and e)). In addition, YAP1 mRNA and protein expression levels were dramatically increased in TNF-α-activated ASM cells compared with those of the blank group (figure 4(f and g)). Collectively, miR-15b-5p could target the YAP1 3 ‘-UTR and inhibit YAP1 expression.

(a) The binding sites between miR-15b-5p and YAP1 mRNA 3 ′ -UTR were predicted by StarBase database. (b and c) Dual-luciferase reporter gene assay was adopted to validate the targeting relationship between miR-15b-5p and the YAP1 mRNA 3’ -UTR. (d-g) The expression levels of YAP1 mRNA and protein in ASM cells transfected with miR-15b-5p mimics or stimulated with TNF-α were detected by RT-qPCR and Western blot. Data are displayed as mean ± standard deviation (n = 3 independent experiments). **P < 0.01 versus mimics-NC group, ***P < 0.001 versus mimics-NC group or blank group.

### YAP1 restoration abrogates the effect of miR-15b-5p on ASM cells

**Figure 5.**
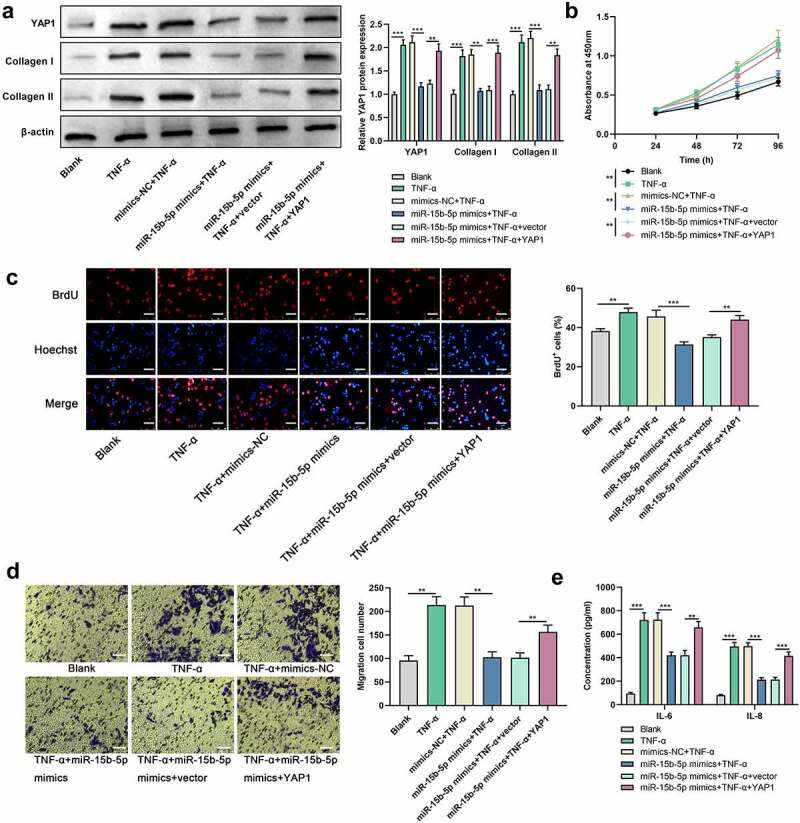
The restoration of YAP1 expression reverses the effect of the upregulated miR-15b-5p on ASM cells.

To investigate whether YAP1 is implicated in miR-15b-5ʹs function in regulating ASM cell proliferation, migration, inflammatory response, and ECM deposition, we co-transfected miR-15b-5p mimics (or mimics-NC) and YAP1 overexpression plasmids (or empty plasmids) into ASM cells. Western blot indicated that the impact of miR-15b-5p overexpression on YAP1, collagen I and collagen III protein expression levels in ASM cells was counteracted by YAP1 overexpression (Figure 5(a)). Additionally, YAP1 overexpression mitigated the impact of miR-15b-5p overexpression on TNF-α-mediated ASM cell viability and migration (Figure 5(b-d)). ELISA indicated that the effect of transfection of miR-15b-5p mimics on TNF-α-activated IL-6 and IL-8 production in ASM cells were dramatically counteracted by YAP1 overexpression (Figure 5(e)). In short, miR-15b-5p impeded TNF-α-induced ASM cell growth, migration, inflammatory response, and ECM deposition by downregulating YAP1 expression.

ASM cells were co-transfected with miR-15b-5p mimics (or mimics-NC) and YAP1 overexpression plasmid (or empty vector) and stimulated with 10 ng/ml of TNF-α for another 24 h. (a) The expression levels of YAP1, collage I, and collage III proteins in ASM cells were detected by Western blot. (b and c) The proliferation of ASM cells was detected by CCK-8 assay and BrdU assay. Scale bar, 250 μm. (d) The migration of ASM cells was detected by Transwell assay. Scale bar, 250 μm. (e) The levels of IL-6 and IL-8 in ASM cells were detected by ELISA. Data are displayed as mean ± standard deviation (n = 3 independent experiments). **P < 0.01 versus blank group or mimics-NC + TNF-α group or miR-15b-5p mimics + TNF-α + vector group, ***P < 0.001 versus blank group or mimics-NC + TNF-α group or miR-15b-5p mimics + TNF-α + vector group.

## Discussion

Airway remodeling in asthma is closely associated with submucosal fibrosis, abnormal proliferation, and migration of ASM cells, and excessive deposition of ECM [22,23]. ASM cell is one of the important sources of inflammatory factors, chemokines, and growth factors [24]. Various cytokines, including TNF-α, transforming growth factor-β (TGF-β), IL-1β, and platelet-derived growth factor (PDGF-BB), have been found to be important mediators of airway remodeling and fibrotic responses, and they can also initiate and drive the inflammatory response in asthma [5,24–26]. As reported, the combined effect of interferon-gamma (IFN-γ), IL-1β, and TNF-α accelerates the release of chemokine CXCL10 in asthmatic ASM cells [27]. Notably, TNF-α stimulation alone can also induce the dysfunction of ASM cells [5,19,28,29]. Here, we found that TNF-α strengthened the proliferative and migrative capabilities of ASM cells; TNF-α could raise the secretion IL-6 and IL-8 of ASM cells, and it increased the expression of ECM-associated proteins (collagen I and collagen III proteins). In other words, TNF-α induces the inflammatory response and ECM deposition in ASM cells, which is consistent with the previous reports [5,19,28,29].

Accumulating research has shown that miRNAs are important modulators in regulating the viability and ECM deposition of asthmatic ASM cells [30,31]. For example, up-regulation of miR-638 targets cyclin D1 and NOR1 and represses PDGF-BB-induced growth and migration of ASM cells [30]. MiR-326 targets TNFSF14 and exacerbates the airway remodeling in asthma by inducing ASM cell proliferation and ECM deposition [31]. MiR-874 inhibits the TNF-α-induced proliferation, migration, inflammation, and ECM deposition in human fetal ASM cells by targeting STAT3 [5]. MiR-15b-5p, as reported, is aberrantly expressed in diverse human cancers [32–34]. Specifically, miR-15b-5p is highly expressed in liver and breast cancer tissues and cell lines, and it can promote the malignant biological processes of tumor cell [32,33]. While in non-small cell lung cancer, miR-15b-5p exerts a tumor-suppressive effect [34]. Here, we discovered that TNF-α stimulation suppressed miR-15b-5p expression in ASM cells. *In-vitro* experiments showed that miR-15b-5p up-regulation could reverse the impact of TNF-α stimulation on the growth and migration of ASM cells, and restrain TNF-α-induced inflammatory response and ECM deposition. Collectively, miR-15b-5p may be a protective modulator in asthma pathogenesis.

As a vital signal transducer of Hippo pathway, YAP1 is extensively expressed in diverse tissues and organs of mammals, and in normal tissues. YAP1 is regulated by other upstream proteins of Hippo pathway such as Lats1/2, Mst1/2, Sav1, and MOB1/2, leading to the cascade amplification [35]. After entering the nucleus, YAP1 can bind to transcriptional factors such as transcriptional enhancer-related domain (TEAD) to function as a transcriptional enhancer, and thus the transcription of downstream genes is promoted [36]. YAP1, as reported, is highly expressed in tumors such as pancreatic ductal adenocarcinoma, colorectal, and bladder cancer, and it expedites the proliferation, migration, and invasion of cancer cells [37–39]. YAP1 is also widely expressed in respiratory epithelial cells, and the Hippo signal pathway is vital in regulating the growth and differentiation of the epithelial cells [40]. As reported, the dysregulation of YAP1 is pertinent to the pathogenesis of asthma [11,18,41]. YAP1 is observed to be up-regulated in bronchial airway tissue of a mouse model with chronic asthma [41] and it can enhance the viability and migrative potential of ASM cells [11]. In addition, YAP1, reportedly, is modulated by multiple miRNAs, such as miR-375, miR-126-5p, miR-582-3p and miR-27b-3p [11,38,42,43]. Here we found that YAP1 was a downstream target of miR-15b-5p in ASM cells; YAP1 was highly expressed in ASM cells stimulated by TNF-α. Here we proved that the impacts of miR-15b-5p up-regulation on ASM cell growth, migration, inflammatory response and ECM deposition could be rescued by YAP1 overexpression. We therefore summarized that miR-15b-5p/YAP1 axis mediated the TNF-α-initiated ASM cell growth, migration, inflammatory response, and ECM deposition.

Notably, there are some limitations of this study: first, only TNF-α-induced ASM cells were used as a model, and whether miR-15b-5p/YAP1 axis is involved in other pro-inflammatory factors-induced dysfunction of ASM cells remains to be explored in the following work; secondly, *in-vivo* studies are lacking in the present work; and finally, other downstream targets of miR-15b-5p need to be identified in the future.

## Conclusion

On all accounts, miR-15b-5p expression is declined in the TNF-α-initiated ASM cells and miR-15b-5p is involved in repressing the proliferation, migration, inflammatory response as well as ECM deposition of ASM cells by targeting YAP1.

## Supplementary Material

Supplemental Material

## Data Availability

The data used to support the findings of this study are available from the corresponding author upon request.
