# Supplementary material for: MicroRNA-15b-5p inhibits tumor necrosis factor alpha-induced proliferation, migration, and extracellular matrix production of airway smooth muscle cells via targeting yes-associated protein 1
Source: Bioengineered. 2022 Feb 16;13(3):5396–406. doi: 10.1080/21655979.2022.2036890 (PMC8974076; doi:10.1080/21655979.2022.2036890)

Supplementary Figure 1 The expression levels of collagen I and collagen III in blank, TNF-α, mimics-NC+TNF-α and miR-15b-5p mimics+TNF-α groups in ASM cells.


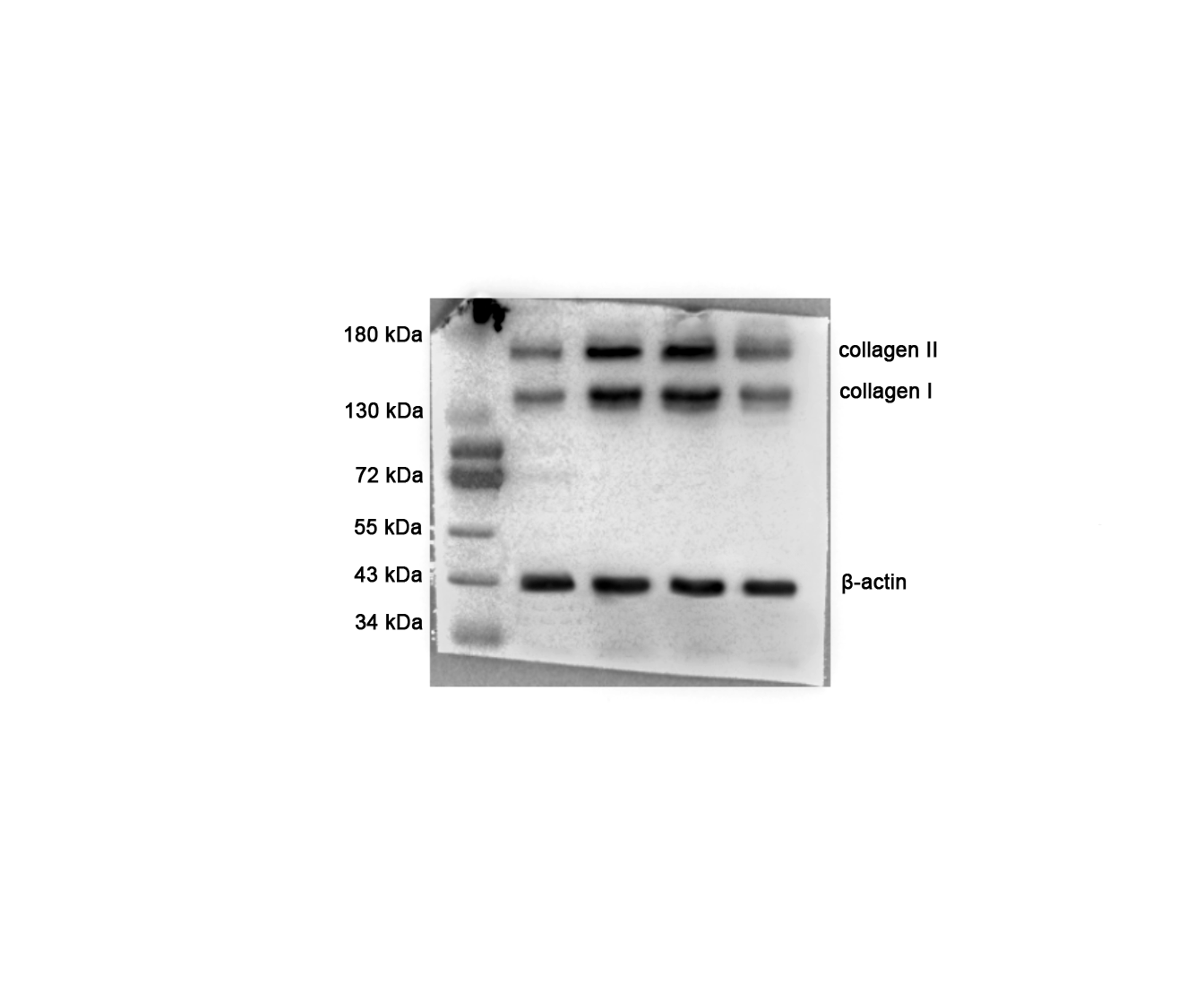

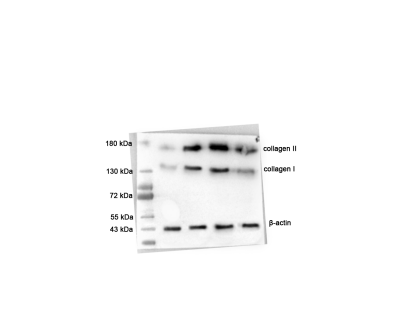

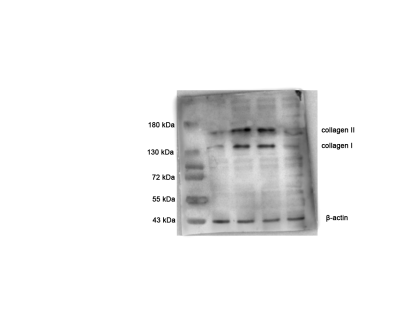


Supplementary Figure 2 The expression of YAP1 protein in ASM cells transfected with miR-15b-5p mimics or mimics-NC.


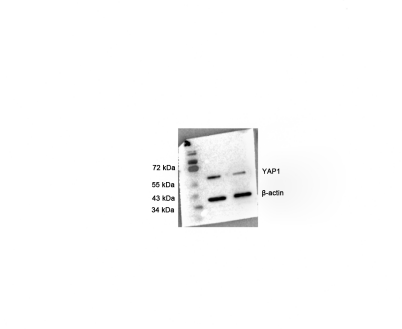

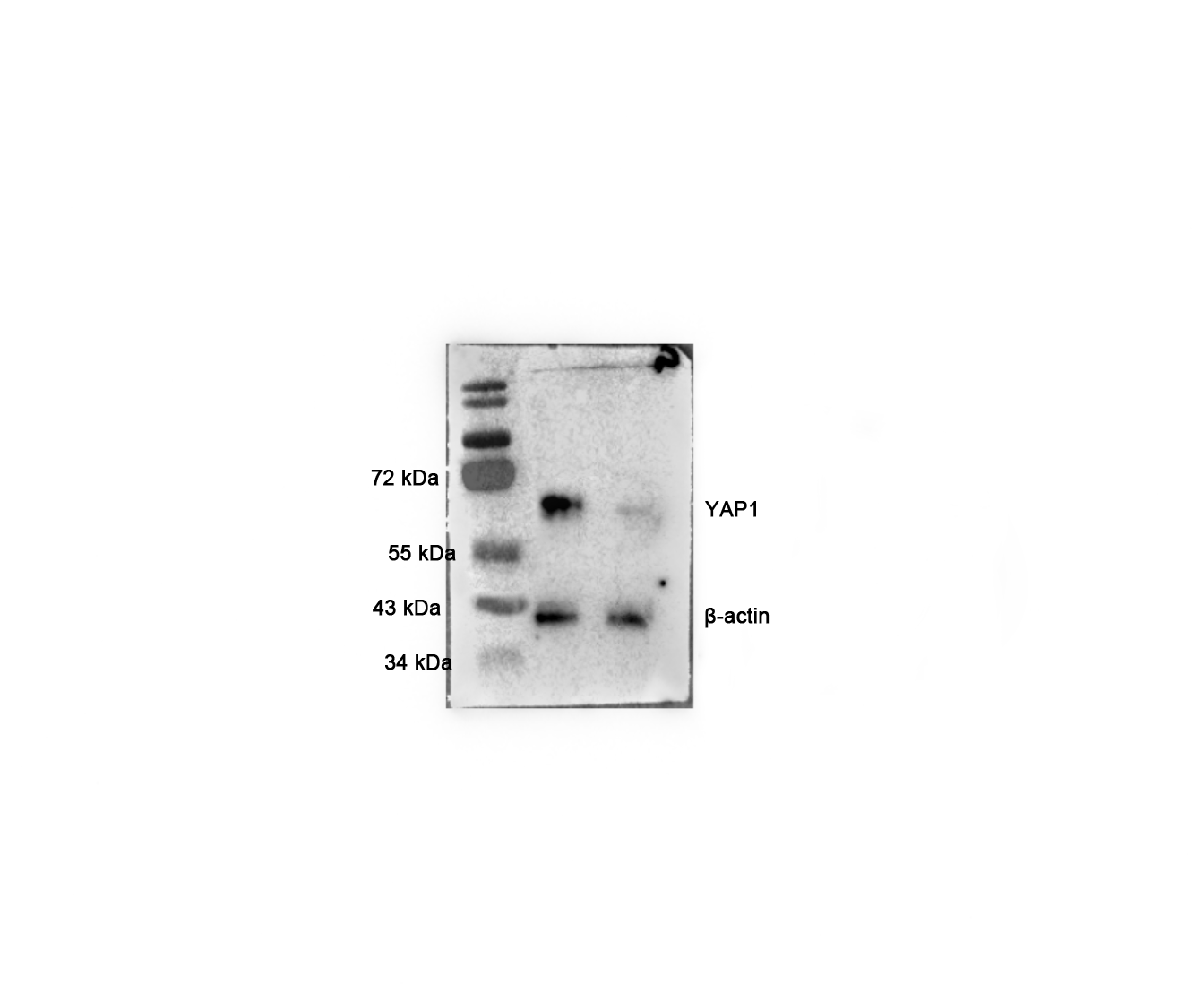

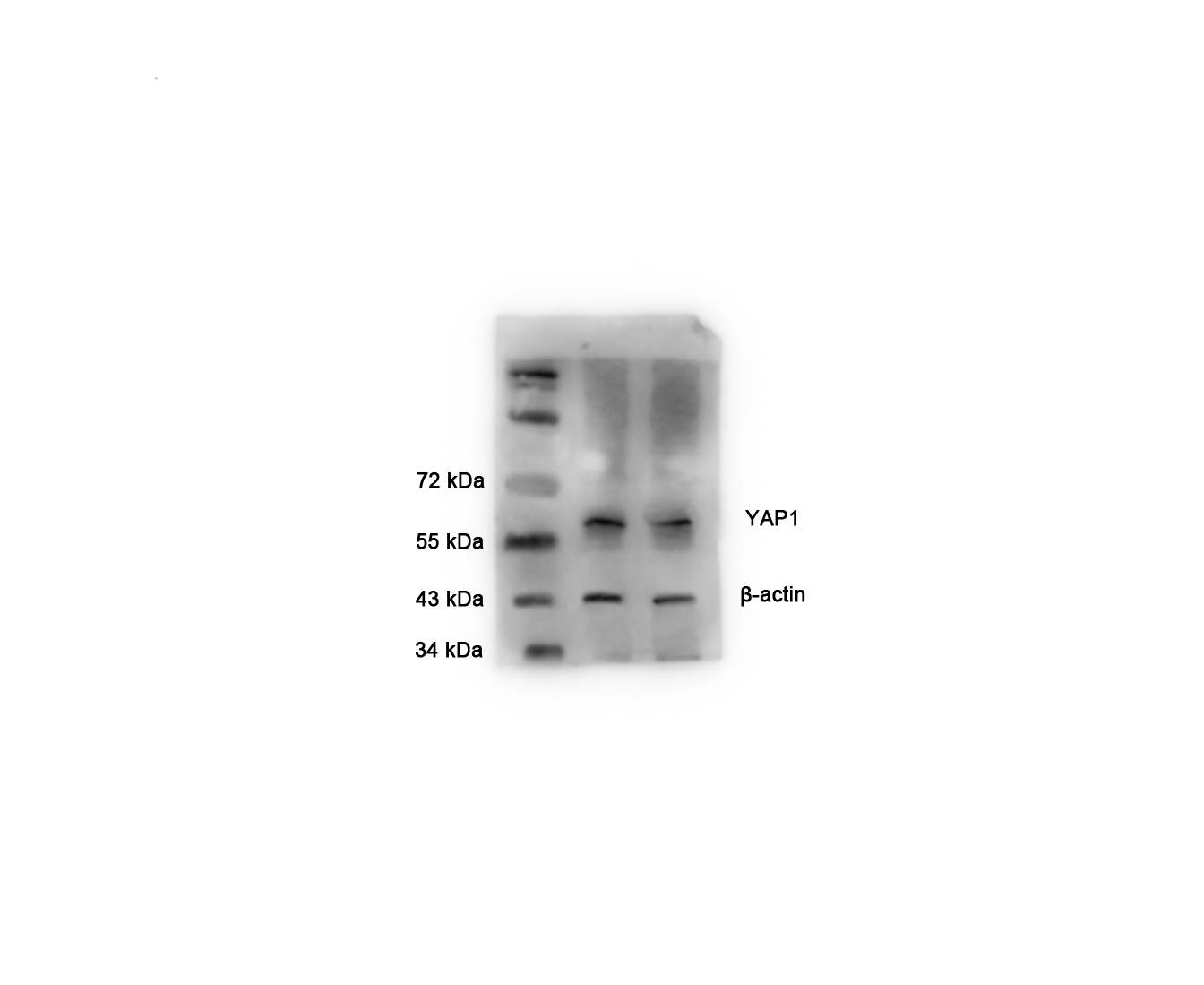


Supplementary Figure 3 The expression of YAP1 protein in ASM cells without or with TNF-α stimulation.


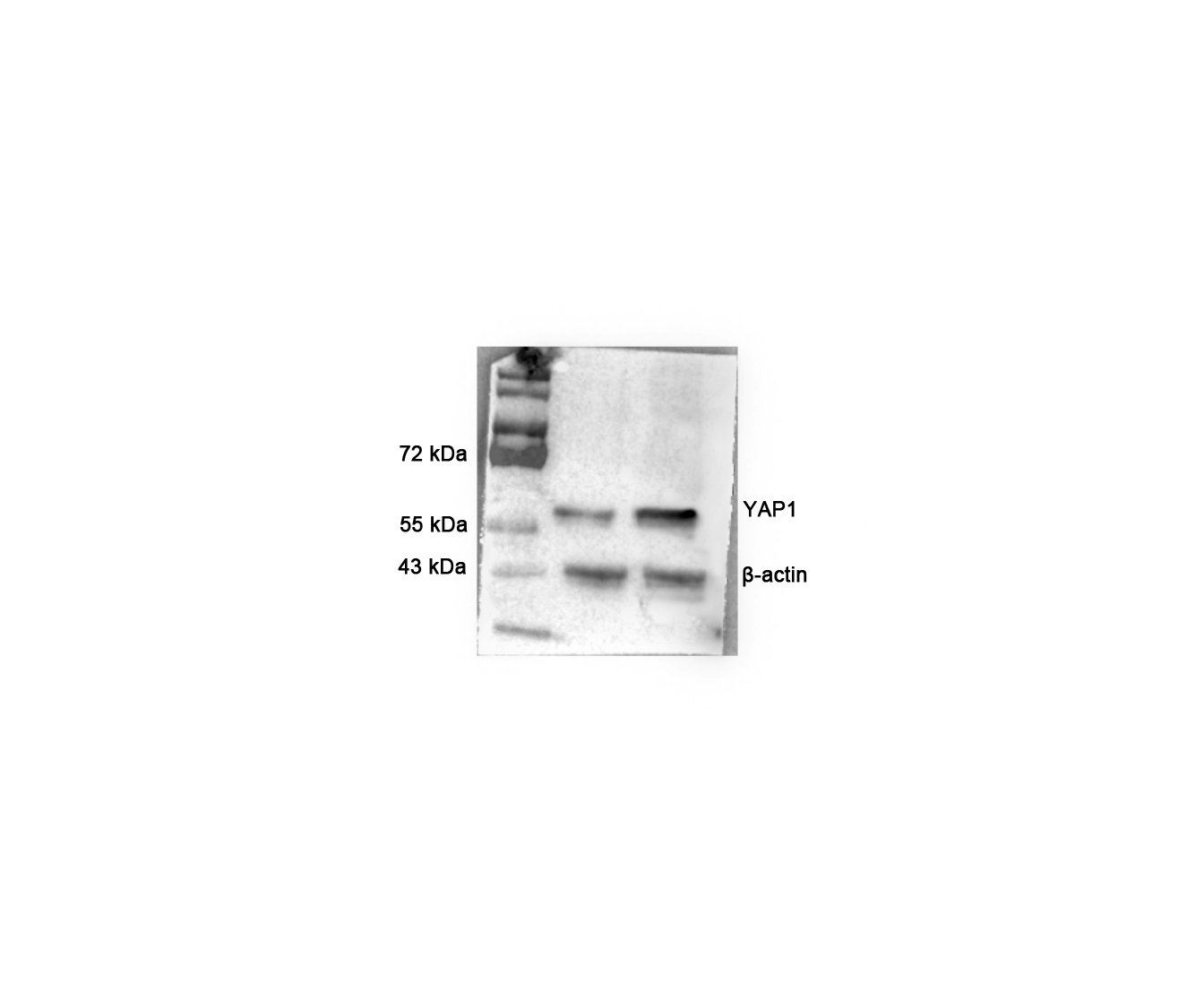

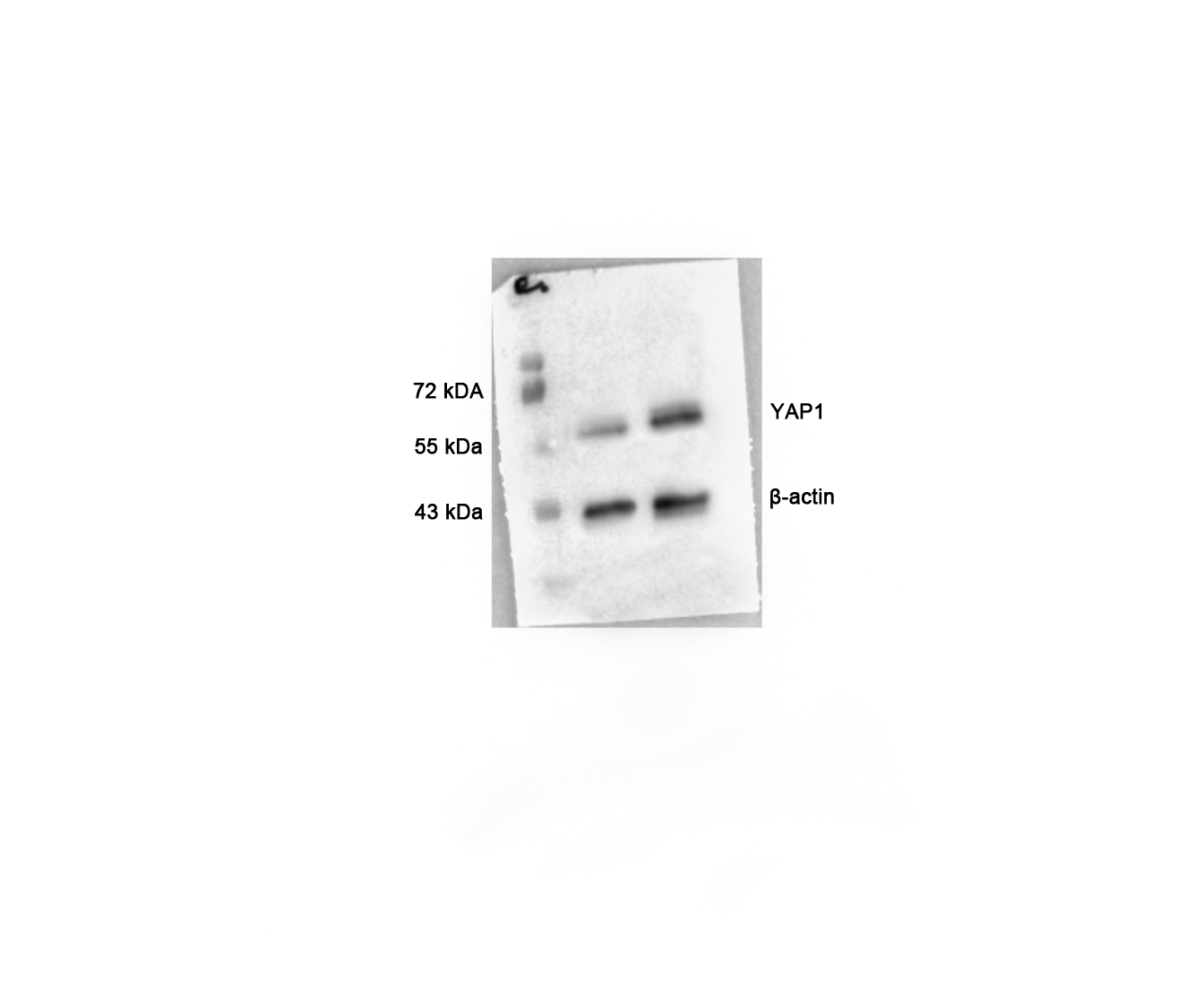

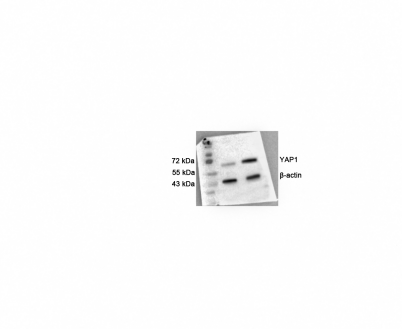


Supplementary Figure 4 The expression levels of YAP1, collagen I and collagen III in blank, TNF-α, mimics-NC+TNF-α, miR-15b-5p mimics+TNF-α, miR-15b-5p mimics+TNF-α+vector and miR-15b-5p mimics+TNF-α+YAP1 groups in ASM cells.


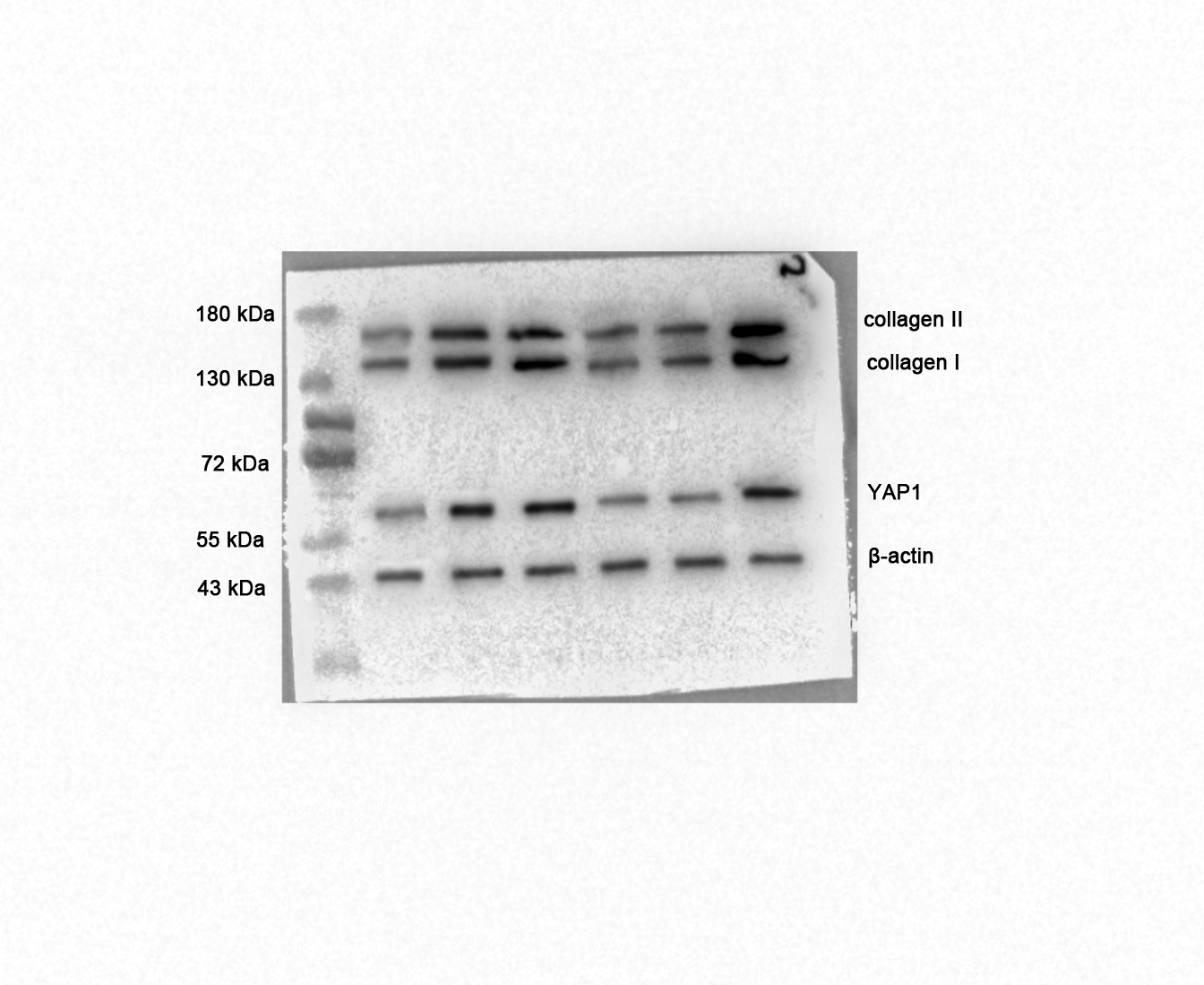

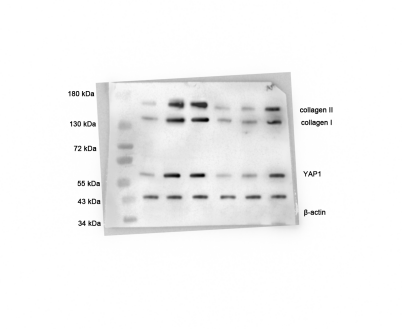

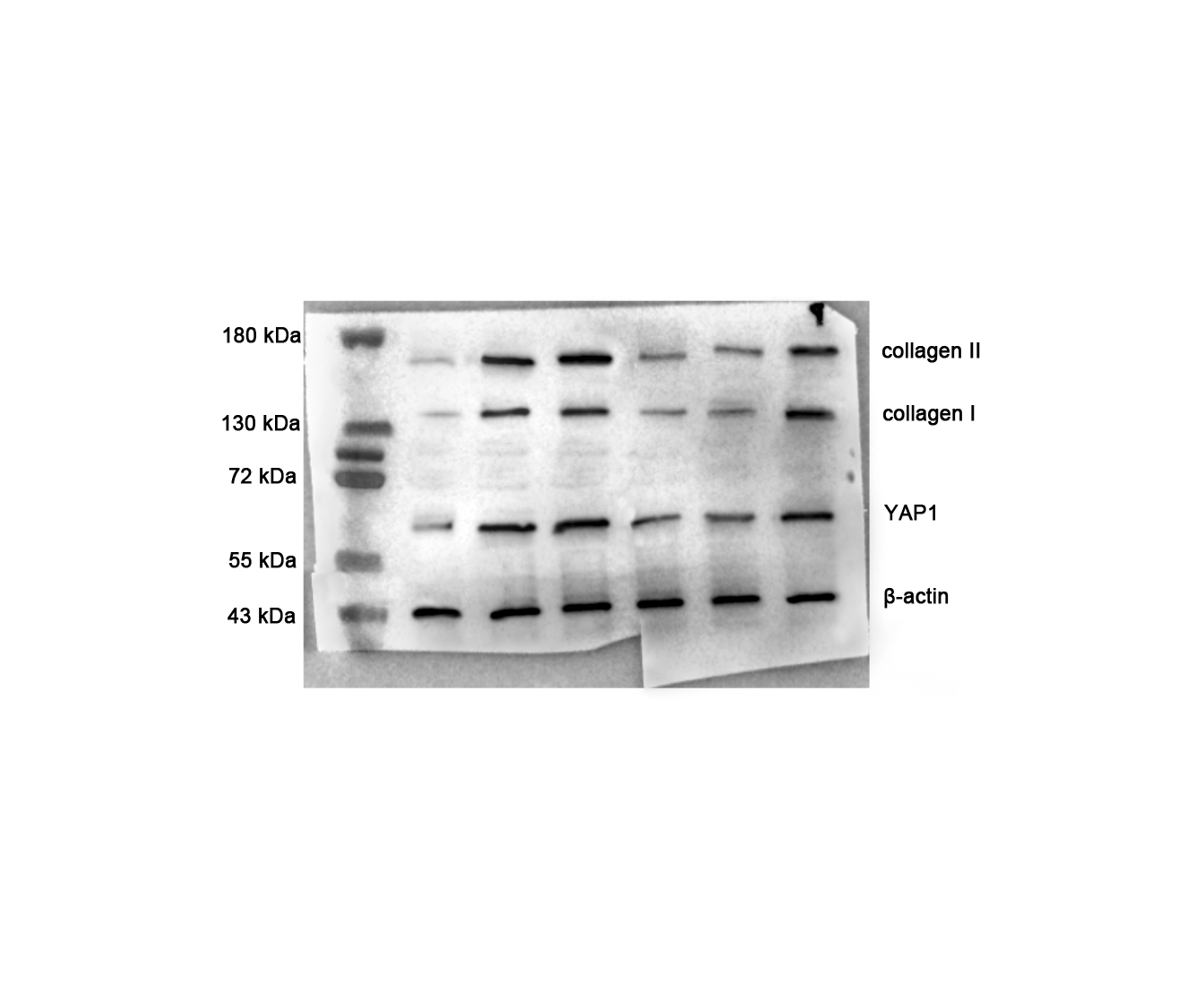

Supplement: Supplemental Material [file KBIE_A_2036890_SM0106.docx]
